# Amphibians and reptiles of the state of San Luis Potosí, Mexico, with comparisons with adjoining states

**DOI:** 10.3897/zookeys.753.21094

**Published:** 2018-04-26

**Authors:** Julio A. Lemos-Espinal, Geoffrey R. Smith, Guillermo A. Woolrich-Piña

**Affiliations:** 1 Laboratorio de Ecología-UBIPRO, FES Iztacala UNAM, Avenida los Barrios 1, Los Reyes Iztacala, Tlalnepantla, edo. de México, México 54090; 2 Department of Biology, Denison University, Granville, OH, USA; 3 Laboratorio de Zoología. División de Biología. Subdirección de Investigación y Posgrado

**Keywords:** Checklist, Chihuahuan Desert, conservation status, herpetofauna, shared species, Sierra Madre Oriental

## Abstract

A summary of the species of amphibians and reptiles of the state has been compiled, including their geographic distributions, habitats, and conservation statuses. The herpetofauna of San Luis Potosí consists of 41 species of amphibians and 141 species of reptiles. San Luis Potosí shares the highest number of species with Hidalgo and Tamaulipas, and the least number of species with Nuevo León. In San Luis Potosí, there are several taxa of particular conservation concern including salamanders, emydid and trionychid turtles, anguid and xenosaurid lizards, and natricid and colubrid snakes.

## Introduction

**Figure 1.**
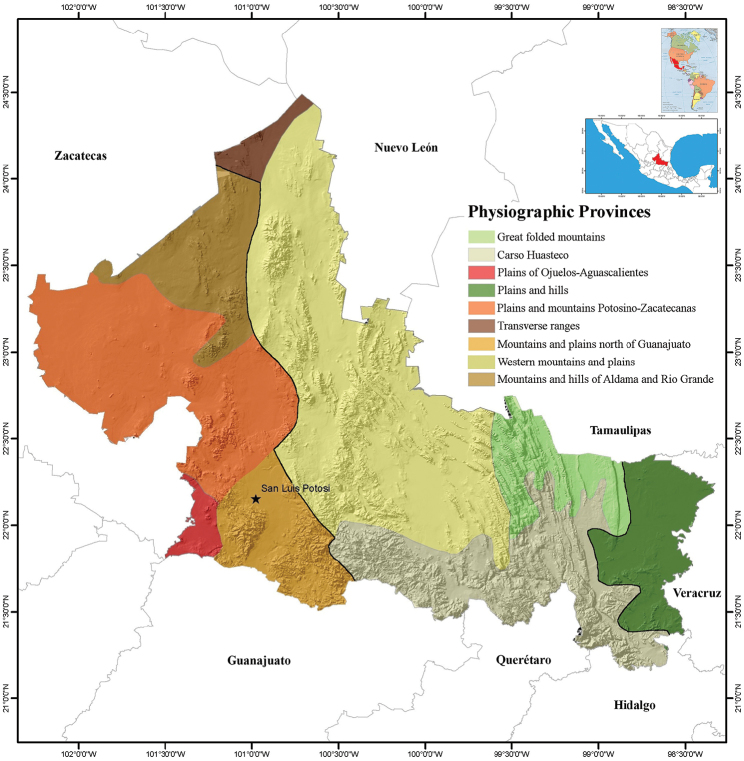
Topographical map with physiographic provinces of the state of San Luis Potosí, Mexico. The thicker black lines delineate the major habitat types found in San Luis Potosí (from west to east): Central Plateau, Sierra Madre Oriental, and North Gulf Coastal Plains (INEGI 2009). Maps modified from Cervantes-Zamora et al. (1990); http://www.gifex.com/fullsize/2009-09-17-3/Mapa-de-Amrica.html; García E – Comisión Nacional para el Conocimiento y Uso de la Biodiversidad (2008).

**Figure 2.**
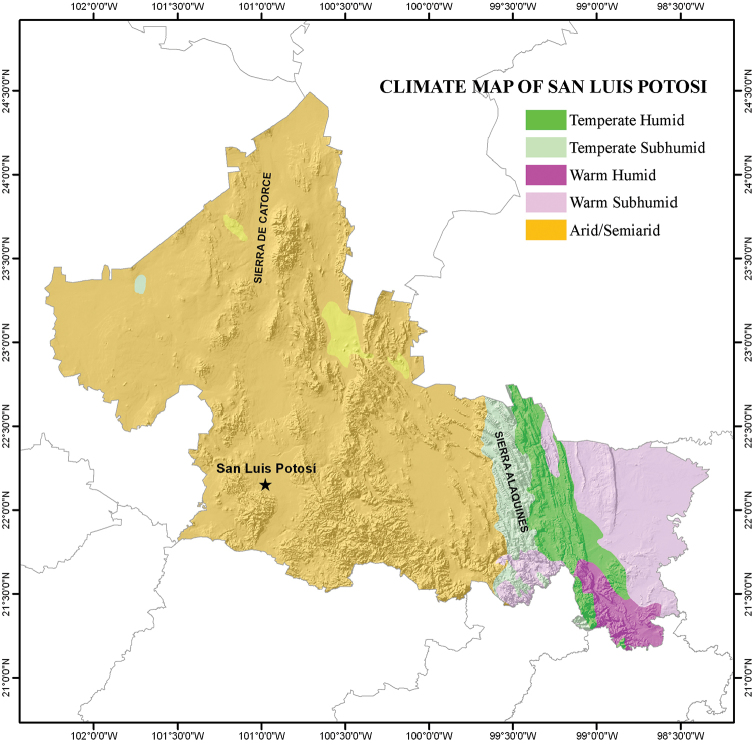
Climate map of the state of San Luis Potosí, Mexico (modified from García – CONABIO 1998).

**Figure 3.**
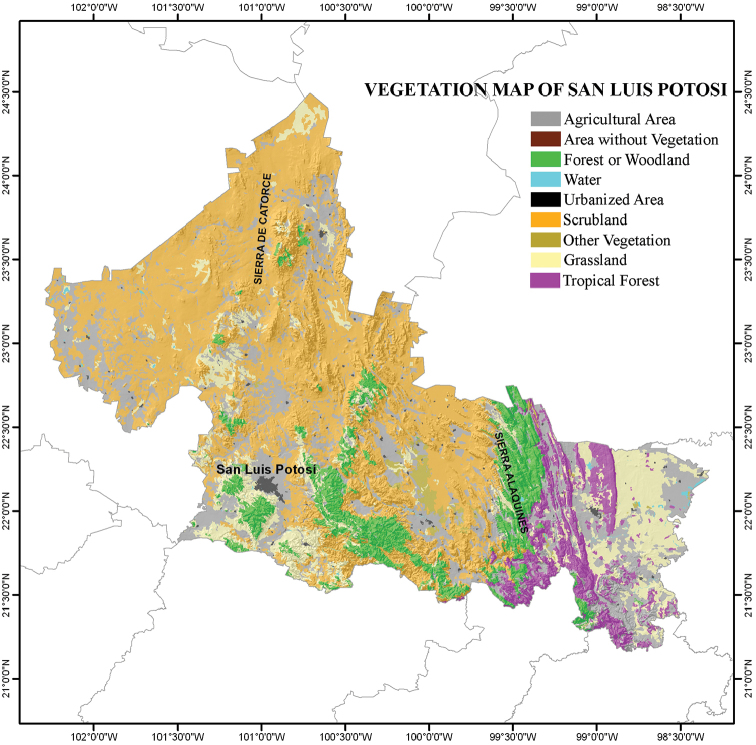
Vegetation type map of the state of San Luis Potosí, Mexico (modified from Dirección General de Geografía – INEGI 2005).

San Luis Potosí is a relatively small state (surface area = 63,068 km^2^, 3.1% of the surface area of Mexico) located in the north-central part of Mexico, between 24°29' and 21°10'N and 98°20' and 102°18'W (see Figure 1; INEGI 2009). The climate of San Luis Potosí varies from the temperate, dry high plains to the warm, relatively humid coast (Lemos-Espinal and Dixon 2013). Several distinctive habitats are found within the boundaries of the state, including the Chihuahuan Desert in the western half and tropical perennial forests in the southeastern portion (= Huasteca Potosina). Three physiographic provinces that vary in their temperature and the moisture retention of their soils (INEGI 2009; Lemos-Espinal and Dixon 2013) are found in San Luis Potosí: the Sierra Madre Oriental, the North Gulf Coastal Plains, and the Central Plateau (Figure 1). The Tropic of Cancer crosses the northern part of the state, and to the east San Luis Potosí nearly reaches the Gulf of Mexico to the east. The elevation of San Luis Potosí varies from about 50 m above sea level to about 3,180 m in Cerro Grande (INEGI 2009). The variation in climate (Figure 2) and physiography of the state have created a mosaic of habitat and vegetation types in San Luis Potosí (Figure 3) that most likely affect the distribution and presence of amphibians and reptiles in the state (see Lemos-Espinal and Dixon 2013 for detailed description of these habitats and vegetation types).

Our understanding of the herpetofauna of San Luis Potosí still remains somewhat limited (see Lemos-Espinal and Dixon 2013 for a review of previous herpetological studies in San Luis Potosí). Our intent with this paper is to encourage others to continue studying the herpetofauna of the state by providing a summary of the species of amphibians and reptiles of the state, their geographic distributions, habitat, and conservation status. By placing all this information into one, easily accessible place, we hope to provide a starting place for further research on the herpetofauna of San Luis Potosí. In addition, a comparison of the amphibian and reptile species lists to those in the neighboring states is provided in an effort to identify unique aspects of the herpetofauna of San Luis Potosí, as well as shared species, with the aim to understand the potential conservation or management needs at the state or regional level.

## Materials and methods

This list of amphibians and reptiles of the state of San Luis Potosí was compiled from the following sources: (1) our own field work; (2) specimens from the Laboratorio de Ecología – UBIPRO (**LEUBIPRO**) collections; (3) a thorough examination of the available literature on amphibians and reptiles of the state; and (4) databases from the Comisión Nacional para el Conocimiento y Uso de la Biodiversidad (National Commission for the Understanding and Use of Biodiversity; **CONABIO**), including records from the following 30 collections:

**AMNH** Collection of Herpetology, Herpetology Department, American Museum of Natural History

**ANSP** Collection of Herpetology, Herpetology Department, Academy of Natural Sciences of Philadelphia

**BMNH** Collection of Herpetology, Zoology Department, The Natural History Museum, British Museum (Natural History)

**CAS** Collection of Herpetology, Herpetology Department, California Academy of Sciences

**CMNH** Collection of Herpetology, Amphibians and Reptiles Section, Carnegie Museum of Natural History – Pittsburgh

**CNAR** Colección Nacional de Anfibios y Reptiles, Instituto de Biología UNAM

**EALC** Ernest A. Liner Collection of Herpetology

**ENCB** Colección Herpetológica, Departamento de Zoología, Escuela Nacional de Ciencias Biológicas

**ENEPI** Colección Herpetológica, Departamento de Biología Experimental, Escuela Nacional de Estudios Profesionales, Unidad Iztacala, UNAM

**FMNH** Division of Amphibians and Reptiles, Field Museum of Natural History

**FSM-UF** Collection of Herpetology, Florida State Museum, University of Florida

**FWMSH** Fort Worth Museum of Sciences and History

**LACM** Collection of Herpetology, Herpetology Section, Natural History Museum of Los Angeles County

**LSUMZ** Collection of Herpetology, Museum of Zoology, Biological Sciences Division, Louisiana State University

**MCZ** Collection of Herpetology, Museum of Comparative Zoology, Harvard University Cambridge

**MNHUK** Museum of Natural History, Division of Herpetology, University of Kansas

**MZFC-UNAM** Colección Herpetológica, Museo de Zoología “Alfonso L. Herrera”, Facultad de Ciencias UNAM

**MVZ** Collection of Herpetology, Museum of Vertebrate Zoology, Division of Biological Sciences, University of California Berkeley

**SDNHM** Collection of Herpetology, Herpetology Department, San Diego Natural History Museum

**TCWC** Collection of Herpetology, Texas Cooperative Wildlife Collection, Texas A&M University

**TNHC** Collection of Herpetology, Texas Natural History Collection, University of Texas Austin

**TU** Collection of Herpetology, Biology Department, Tulane University, New Orleans

**UAZ** Amphibians and Reptiles Collection, University of Arizona

**UCM** Collection of Herpetology, University of Colorado Museum

**UIMNH** Collection of Herpetology, University of Illinois Museum of Natural History

**UIUC** Collection of Herpetology, Museum of Natural History, University of Illinois at Urbana-Champaign

**UMMZ** Collection of Herpetology, Museum of Zoology, University of Michigan Ann Arbor

**USNM** Collection of Herpetology, Department of Vertebrate Zoology, National Museum of Natural History, Smithsonian Institution

**UTAMM** Merriam Museum, University of Texas Arlington

**UTEP** Collection of Herpetology, Laboratory of Environmental Biology, Biological Sciences Department, University of Texas - El Paso

Amphibian names follow Frost (2017) and AmphibiaWeb (2017) (http://amphibiaweb.org) and reptile names follow Uetz and Hošek (2017). Species were included in the list if they had confirmed records, either by direct observation or through documented museum records or vouchers. Species accumulation curves were created for the total herpetofauna, amphibians, and reptiles using the year of the first recorded observation for each species. Such species accumulation curves are likely to serve as good estimators of the potential species richness of amphibians and reptiles (see Raxworthy et al. 2012). In addition, the conservation status of each species was recorded based on three sources: 1) the IUCN Red List 2017; 2) Environmental Vulnerability Scores from Wilson et al. (2013a,b) and Johnson et al. (2015); 3) listing in SEMARNAT (2010).

The number of overlapping species with those neighboring states for which a recent checklist exists (Hidalgo: Lemos-Espinal and Smith 2015, Lemos-Espinal and Dixon 2016; Nuevo León: Lemos-Espinal et al. 2016; Tamaulipas: Farr 2015, Terán-Juárez et al. 2016; Querétaro: Dixon and Lemos-Espinal 2010) was determined, and hierarchical clustering analyses with single linkage and Euclidean distances using Systat 13 software (SYSTAT software, Chicago, IL) used to examine the similarities among the herpetofaunas of San Luis Potosí and its neighboring states (see Enderson et al. 2009 for a similar analysis). Lists were updated for Hidalgo (substituting *Lampropeltis polyzona* for *L. triangulum*, Ruane et al. 2014, Uetz and Hošek 2017); Nuevo León (adding *Chiropterotriton miquihuanus*, Campbell et al. 2014; *Crotalus morulus*, Bryson et al. 2014, Uetz and Hošek 2017; and substituting *Lampropeltis annulata* for *L. triangulum*, Ruane et al. 2014, Uetz and Hošek 2017); Querétaro (substituting substituting *L. annulata* for *L. triangulum*, Ruane et al. 2014, Uetz and Hošek 2017; adding *Amastridium sapperi*, Calzada-Arciniega 2014). The neighboring states of Guanajuato, Veracruz, and Zacatecas do not have recent, comprehensive checklists of amphibians and reptiles available so were not included in comparisons.

## Results and discussion

San Luis Potosí is home to 182 species of amphibians and reptiles which represent 33 families and 98 genera (Table 1). These include 41 species of amphibians (six salamanders, 35 anurans) and 141 of reptiles (one crocodilian, seven turtles, 48 lizards, 85 snakes). The herpetofaunal account for the state published by Lemos-Espinal and Dixon (2013) listed a total of 181 species of amphibians and reptiles, including Dennis’ Chirping Frog (*Eleutherodactylus dennisi*), a species not included in this paper since the only record for this species (ENCB-14250 – *E. dennisi*; collected on August 13^th^, 1989. 1 km N of Apetzco, municipality of Xilitla, SLP), seems to be a misidentified *E. longipes. Amastridium sapperi* was added based on Calzada-Arciniega (2014). *Sceloporus cowlesi* was substituted for *S. consobrinus* based on Leaché (pers. comm.), *Scincella silvicola* for *S. caudaequinae* based on Linkem et al. (2011) and Uetz and Hošek (2017), and *Holcosus undulatus* for *H. amphigrammus* based on Meza-Lázaro and Nieto-Montes de Oca (2015). *Lampropeltis triangulum* was substituted for *L. annulata* and *L. polyzona* based on Ruane et al. (2014) and Uetz and Hošek (2017). *Xenosaurus newmanorum* was regarded as endemic to Mexico but not to San Luis Potosí based on Nieto Montes de Oca et al. (2017). No species is endemic to the state, and four are introduced: the American Bullfrog (*Rana catesbeiana*), the Common Four-clawed Gecko (*Gehyra mutilata*), the Common House Gecko (*Hemidactylus frenatus*), and the Mediterranean House Gecko (*H. turcicus*).

A list of 17 species (nine amphibians, eight reptiles) potentially occurring in San Luis Potosí was compiled (Table 2), based on species for which undocumented observations in San Luis Potosí exist but for which museum or other records are not available, and on species that have not been recorded or observed in the state, but whose distributional ranges come close to the borders of San Luis Potosí.

**Figure 4.**
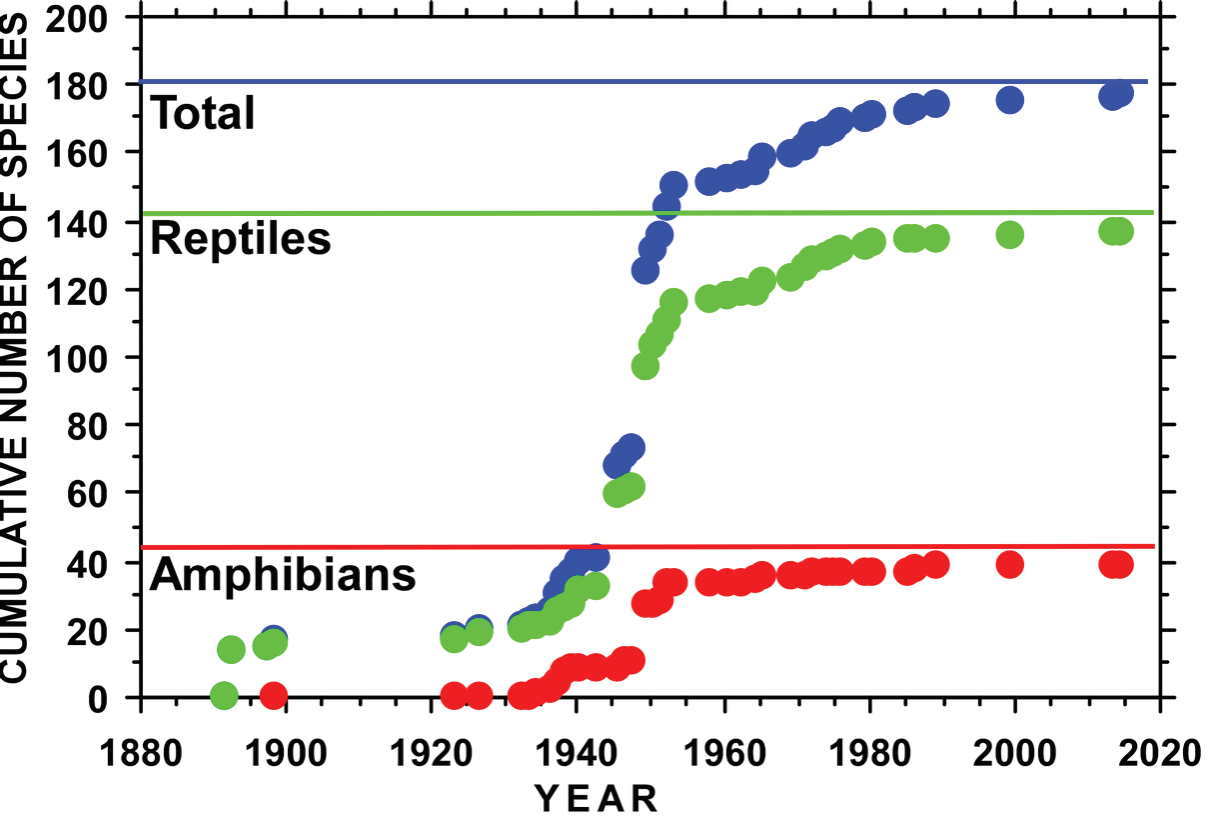
Species accumulation curves for the total herpetofauna, amphibians, and reptiles from San Luis Potosí. Horizontal lines are estimated asymptotes for the species accumulation curves.

The species accumulation curves for all species, amphibians, and reptiles suggest that the current list of species is close to being the likely species richness for San Luis Potosí (Figure 4). These curves show a dramatic increase in documents herpetofaunal species during the 1940’s and 1950’s, primarily associated with the work of Edward Taylor and Hobart Smith (Smith 1939, Smith and Taylor 1945, 1950; Taylor 1949, 1950, 1952, 1953). Taken together with the relatively limited number of potential additions to the herpetofauna of San Luis Potosí (see Table 2), it seems likely that, barring the discovery of multiple cryptic species, that this is a fairly complete list of the herpetofauna of San Luis Potosí.

### General distribution

Seventeen of the 41 species of Amphibians that inhabit San Luis Potosí are endemic to Mexico, two of which are restricted to small areas in the Sierra Madre Oriental around southeastern San Luis Potosí (Table 1). Eight more are distributed mainly in eastern Mexico (Table 1). The remaining seven endemic amphibians are widely distributed in central, eastern, and even western Mexico (Table 1). Of the 24 amphibian species not endemic to Mexico that inhabit San Luis Potosí, one is an introduced species, eleven more are found in the United States and Mexico, the remaining 12 species have a wide distribution from Canada to Central America, from the United States to Central or South America, or from Mexico to Central or South America (Table 1).

Morelet’s Crocodile (*Crocodylus moreletii*), is widely distributed from Tamaulipas to Central America. Three of the seven species of turtles that inhabit San Luis Potosí are endemic to Mexico, two of them to eastern Mexico and another is widely distributed in western and central Mexico (Table 1). The four non-endemic species of turtles are found from southern Canada to the Balsas River of Guerrero, from the United States to Mexico, or from Mexico to South America (Table 1). Twenty-five of the 48 species of lizards that occur in the state are endemic to Mexico, two more have a narrow distribution in northern San Luis Potosí and southern Tamaulipas (*Ophisaurus incomptus* and *Lepidophyma micropholis*) one is found from southern Tamaulipas to northern Hidalgo (*Xenosaurus newmanorum*), another to a small area in Coahuila, Nuevo León, and San Luis Potosí (*Sceloporus goldmani*), one more to northern Querétaro and adjacent San Luis Potosí (*Lepidophyma occulor*), and another to a small area in San Luis Potosí, Querétaro, and Hidalgo (*Lepidophyma gaigeae*). Most of the remaining 19 lizards endemic to Mexico are distributed mainly in eastern or central Mexico, in northern Mexico (*Holbrookia approximans*), or in western and eastern Mexico (Table 1). The remaining 23 species of lizards that inhabit San Luis Potosí are not endemic to Mexico; 13 of the non-endemic are species found in the United States and Mexico; two are found from southern United States to Central America; five are distributed from Mexico to Central America; and three are introduced to San Luis Potosí (Table 1). Twenty-eight of the 85 species of snakes are endemic to Mexico (Table 1). Twenty-three snake species that are found in San Luis Potosí are distributed from the United States to Mexico; another 22 species range from Mexico to Central or even South America; eight more species are found from central or southern United States to Central or South America; and four more range from Canada to Mexico or even Central America (Table 1).

**Table 1.** Checklist of amphibians and reptiles of San Luis Potosí providing Global Distribution (0 = Introduced; 1 = Endemic to Mexico; 2 = Distributed in the United States and Mexico; 3 = Distributed from Mexico and south of Mexico; 4 = Distributed from the United States to Central or even South America; 5 = Distributed from Canada to Mexico or south of Mexico), the habitat type (CD = Chihuahuan Desert, SMO = Temperate Forests of the Sierra Madre Oriental, SBT = Subtropics of the Sierra Madre Oriental; GEN = Generalist – occupies more than one habitat type), IUCN Status (DD = Data Deficient; LC = Least Concern, VU = Vulnerable, NT = Near Threatened; EN = Endangered; CE = Critically Endangered; NL = not listed), population trend (+ = Increasing, = = Stable, - = Decreasing, ? = Unknown) according to the IUCN Red List (The IUCN Red List of Threatened Species, Version 2016.3; www.iucnredlist.org; accessed 1 March 2017), Environmental Vulnerability Score (EVS; the higher the score the greater the vulnerability; NE = not evaluated) from Wilson et al. (2013a,b) and Johnson et al. (2015a), and conservation status in Mexico according to SEMARNAT (2010) (P = in danger of extinction, A = threatened; Pr = subject to special protection, NL – not listed). Source denotes whether the species was observed in the field by the authors (A), documented in the CONABIO data base and/or museum collections (C/M), or found in the literature (citation of source). N/A = not applicable due to being non-native.

Taxa	GD	Habitat type	IUCN	Population Trend	EVS	SEMARNAT	Source
**CLASS AMPHIBIA**							
**ORDER CAUDATA**							
**Ambystomatidae (1 genus, 1 species)**							
*Ambystoma velasci* (Dugès, 1888)	1	**CD**	**LC**	?	**10**	**Pr**	**A**
**Plethodontidae (3 genera, 4 species)**							
*Bolitoglossa platydactyla* (Gray, 1831)	1	**SBT**	**NT**	–	**15**	**Pr**	**C/M**
*Chiropterotriton magnipes* Rabb, 1965	1	**SMO**	**CE**	–	**16**	**Pr**	**C/M**
*Chiropterotriton multidentatus* (Taylor, 1938)	1	**SMO**	**EN**	–	**15**	**Pr**	**C/M**
*Isthmura bellii* (Gray, 1850)	1	**SMO**	**VU**	–	**12**	**A**	**C/M**
**Salamandridae (1 genus, 1 species)**							
*Notophthalmus meridionalis* (Cope, 1880)	2	**SBT**	**EN**	–	**12**	**Pr**	**C/M**
**ORDER ANURA**							
**Bufonidae (3 genera, 6 species)**							
*Anaxyrus cognatus* (Say, 1823)	5	**CD**	**LC**	?	**9**	**NL**	**A**
*Anaxyrus debilis* (Girard, 1854)	2	**CD**	**LC**	=	**7**	**Pr**	**A**
*Anaxyrus punctatus* Baird & Girard, 1852	2	**CD**	**LC**	=	**5**	**NL**	**A**
*Incilius nebulifer* Girard, 1854	2	**GEN**	**LC**	=	**6**	**NL**	**A**
*Incilius occidentalis* Camerano, 1879	1	**CD**	**LC**	=	**11**	**NL**	**A**
*Rhinella horribilis* (Linnaeus, 1758)	4	**GEN**	**NL**	?	**NE**	**NL**	**A**
**Craugastoridae (1 genus, 3 species)**							
*Craugastor augusti* (Dugès, 1879)	2	**SMO**	**LC**	=	**8**	**NL**	**A**
*Craugastor berkenbuschii* (Peters, 1870)	1	**CD**	**NT**	–	**14**	**Pr**	**C/M**
*Craugastor decoratus* (Taylor, 1942)	1	**SMO**	**VU**	?	**15**	**Pr**	**C/M**
**Eleutherodactylidae (1 genus, 5 species)**							
*Eleutherodactylus cystignathoides* (Cope, 1878)	2	**GEN**	**LC**	–	**12**	**NL**	**A**
*Eleutherodactylus guttilatus* (Cope, 1870)	2	**GEN**	**LC**	?	**11**	**NL**	**C/M**
*Eleutherodactylus leprus* (Cope, 1879)	3	**SMO**	**VU**	–	**12**	**NL**	**C/M**
*Eleutherodactylus longipes* (Baird, 1869)	1	**SBT**	**VU**	?	**15**	**NL**	**C/M**
*Eleutherodactylus verrucipes* Cope, 1865	1	**GEN**	**VU**	–	**16**	**Pr**	**C/M**
**Hylidae (7 genera, 9 species)**							
*Hyla arenicolor* Cope, 1886	2	**GEN**	**LC**	=	**7**	**NL**	**A**
*Hyla eximia* Baird, 1854	1	**GEN**	**LC**	=	**10**	**NL**	**A**
*Hyla plicata* Brocchi, 1877	1	**SMO**	**LC**	=	**11**	**A**	**C/M**
*Rheohyla miotympanum* (Cope, 1863)	1	**SBT**	**NT**	–	**9**	**NL**	**A**
*Sarcohyla arborescandens* (Taylor, 1939)	1	**SBT**	**EN**	–	**11**	**Pr**	**C/M**
*Scinax staufferi* (Cope, 1865)	3	**GEN**	**LC**	=	**4**	**NL**	**A**
*Smilisca baudinii* (Duméril & Bibron, 1841)	4	**SBT**	**LC**	=	**3**	**NL**	**A**
*Tlalocohyla picta* (Günther, 1901)	3	**SBT**	**LC**	+	**8**	**NL**	**A**
*Trachycephalus typhonius* (Linnaeus, 1758)	3	**SBT**	**LC**	=	**4**	**NL**	**A**
**Leptodactylidae (1 genus, 2 species)**							
*Leptodactylus fragilis* (Brocchi, 1877)	4	**SBT**	**LC**	=	**5**	**NL**	**A**
*Leptodactylus melanonotus* (Hallowell, 1861)	3	**SBT**	**LC**	=	**6**	**NL**	**A**
**Microhylidae (2 genera, 2 species)**							
*Gastrophryne olivacea* (Hallowell, 1857)	2	**SBT**	**LC**	=	**9**	**Pr**	**C/M**
*Hypopachus variolosus* (Cope, 1866)	4	**GEN**	**LC**	=	**4**	**NL**	**A**
**Ranidae (1 genus, 5 species)**							
*Rana berlandieri* Baird, 1859	4	**GEN**	**LC**	=	**7**	**Pr**	**A**
*Rana catesbeiana* Shaw, 1802	0	**N/A**	**N/A**	**N/A**	**N/A**	**N/A**	**C/M**
*Rana johni* Blair, 1965	1	**SBT**	**EN**	–	**14**	**Pr**	**C/M**
*Rana montezumae* Baird, 1854	1	**GEN**	**LC**	–	**13**	**Pr**	**A**
*Rana neovolcanica* Hillis & Frost, 1985	1	**CD**	**NT**	–	**13**	**A**	**A**
**Rhinophrynidae (1 genus, 1 species)**							
*Rhinophrynus dorsalis* Duméril & Bibron, 1841	4	**SBT**	**LC**	=	**8**	**Pr**	**A**
**Scaphiopodidae (2 genera, 2 species)**							
*Scaphiopus couchii* Baird, 1854	2	**GEN**	**LC**	=	**3**	**NL**	**A**
*Spea multiplicata* (Cope, 1863)	2	**GEN**	**LC**	=	**6**	**NL**	**A**
**CLASS REPTILIA**							
**ORDER CROCODYLIA**							
**Crocodylidae (1 genus, 1 species)**							
*Crocodylus moreletii* Duméril & Bibron, 1851	3	**SBT**	**LC**	=	**13**	**Pr**	**C/M**
**ORDER TESTUDINES**							
**Emydidae (2 genera, 2 species)**							
*Terrapene mexicana* (Gray, 1849)	1	**SBT**	**NL**	?	**19**	**NL**	**C/M**
*Trachemys venusta* (Gray, 1855)	3	**GEN**	**NL**	?	**13**	**NL**	**C/M**
**Kinosternidae (1 genus, 4 species)**							
*Kinosternon herrerai* Stejneger, 1925	1	**GEN**	**NT**	–	**14**	**Pr**	**A**
*Kinosternon hirtipes* (Wagler, 1830)	2	**GEN**	**LC**	–	**10**	**Pr**	**A**
*Kinosternon integrum* LeConte, 1854	1	**GEN**	**LC**	=	**11**	**Pr**	**A**
*Kinosternon scorpioides* (Linnaeus, 1766)	3	**SBT**	**NL**	?	**10**	**Pr**	**A**
**Trionychidae (1 genus, 1 species)**							
*Apalone spinifera* (Lesueur, 1827)	5	**SBT**	**LC**	–	**15**	**Pr**	**C/M**
**ORDER SQUAMATA**							
**SUBORDER LACERTILIA**							
**Anguidae (4 genera, 5 species)**							
*Abronia taeniata* (Wiegmann, 1828)	1	**GEN**	**VU**	–	**15**	**Pr**	**A**
*Barisia ciliaris* (Smith, 1942)	1	**GEN**	**NL**	?	**15**	**NL**	**A**
*Gerrhonotus infernalis* Baird, 1859	2	**GEN**	**LC**	=	**13**	**NL**	**A**
*Gerrhonotus ophiurus* Cope, 1866	1	**GEN**	**LC**	?	**12**	**NL**	**A**
*Ophiosaurus incomptus* (McConkey, 1955)	1	**SBT**	**DD**	?	**15**	**Pr**	**C/M**
**Corytophanidae (2 genera, 2 species)**							
*Corytophanes hernandesii* (Wiegmann, 1831)	3	**SBT**	**LC**	=	**13**	**Pr**	**A**
*Laemanctus serratus* Cope, 1864	3	**SBT**	**LC**	=	**8**	**Pr**	**A**
**Crotaphytidae (1 genus, 1 species)**							
*Crotaphytus collaris* (Say, 1823)	2	**CD**	**LC**	=	**13**	**A**	**A**
**Dactyloidae (1 genus, 2 species)**							
*Anolis petersii* Bocourt, 1873	3	**SBT**	**NL**	?	**9**	**NL**	**C/M**
*Anolis sericeus* Hallowell, 1856	3	**SBT**	**NL**	?	**8**	**NL**	**A**
**Dibamidae (1 genus, 1 species)**							
*Anelytropsis papillosus* Cope, 1885	1	**GEN**	**LC**	–	**10**	**A**	**C/M**
**Eublepharidae (1 genus, 1 species)**							
*Coleonyx elegans* Gray, 1845	3	**SBT**	**LC**	=	**9**	**A**	**C/M**
**Gekkonidae (2 genera, 3 species)**							
*Gehyra mutilata* (Wiegmann, 1834)	0	**N/A**	**N/A**	**N/A**	**N/A**	**N/A**	**C/M**
*Hemidactylus frenatus* Schlegel, 1836	0	**N/A**	**N/A**	**N/A**	**N/A**	**N/A**	**A**
*Hemidactylus turcicus* (Linnaeus, 1758)	0	**N/A**	**N/A**	**N/A**	**N/A**	**N/A**	**A**
**Iguanidae (1 genus, 1 species)**							
*Ctenosaura acanthura* (Shaw, 1802)	1	**SBT**	**NL**	?	**12**	**Pr**	**A**
**Phrynosomatidae (4 genera, 19 species)**							
*Cophosaurus texanus* Troschel, 1852	2	**CD**	**LC**	=	**14**	**A**	**A**
*Holbrookia approximans* Baird, 1859	1	**CD**	**NL**	?	**14**	**NL**	**A**
*Phrynosoma cornutum* (Harlan, 1824)	2	**CD**	**LC**	=	**11**	**NL**	**A**
*Phrynosoma modestum* Girard, 1852	2	**CD**	**LC**	=	**12**	**NL**	**A**
*Phrynosoma orbiculare* (Linnaeus, 1758)	1	**GEN**	**LC**	=	**12**	**A**	**A**
*Sceloporus cautus* Smith, 1938	1	**GEN**	**LC**	–	**15**	**NL**	**A**
*Sceloporus cowlesi* Lowe & Norris, 1956	2	**CD**	**NL**	?	**13**	**NL**	**A**
*Sceloporus dugesii* Bocourt, 1873	1	**CD**	**LC**	=	**13**	**NL**	**A**
*Sceloporus goldmani* Smith, 1937	1	**CD**	**EN**	–	**15**	**NL**	**C/M**
*Sceloporus grammicus* Wiegmann, 1828	2	**GEN**	**LC**	=	**9**	**Pr**	**A**
*Sceloporus minor* Cope, 1885	1	**CD**	**LC**	=	**14**	**NL**	**A**
*Sceloporus olivaceus* Smith, 1934	2	**CD**	**LC**	=	**13**	**NL**	**A**
*Sceloporus parvus* Smith, 1934	1	**GEN**	**LC**	=	**15**	**NL**	**A**
*Sceloporus poinsettii* Baird & Girard, 1852	2	**CD**	**LC**	=	**12**	**NL**	**A**
*Sceloporus scalaris* Wiegmann, 1828	1	**CD**	**LC**	=	**12**	**NL**	**A**
*Sceloporus serrifer* Cope, 1866	4	**GEN**	**LC**	=	**6**	**NL**	**A**
*Sceloporus spinosus* Wiegmann, 1828	1	**CD**	**LC**	=	**12**	**NL**	**A**
*Sceloporus torquatus* Wiegmann, 1828	1	**GEN**	**LC**	=	**11**	**NL**	**A**
*Sceloporus variabilis* Wiegmann, 1828		**GEN**	**LC**	=	**5**	**NL**	**A**
**Scincidae (2 genera, 5 species)**							
*Plestiodon dicei* (Ruthven & Gaige, 1933)	1	**SMO**	**NL**	?	**7**	**NL**	**A**
*Plestiodon lynxe* (Wiegmann, 1834)	1	**GEN**	**LC**	=	**10**	**Pr**	**A**
*Plestiodon obsoletus* Baird & Girard, 1852	2	**CD**	**LC**	=	**11**	**NL**	**C/M**
*Plestiodon tetragrammus* Baird, 1859	2	**GEN**	**LC**	=	**12**	**NL**	**A**
*Scincella caudaequinae* (Smith, 1951)	1	**GEN**	**NL**	?	**NE**	**NL**	**A**
**Teiidae (2 genera, 3 species)**							
*Aspidoscelis gularis* (Baird & Girard, 1852)	2	**CD**	**LC**	=	**9**	**NL**	**A**
*Aspidoscelis inornatus* (Baird, 1859)	2	**CD**	**LC**	–	**14**	**NL**	**A**
*Holcosus amphigrammus* (Smith & Laufe, 1945)	1	**GEN**	**NL**	?	**12**	**NL**	**A**
**Xantusiidae (1 genus, 4 species)**							
*Lepidophyma gaigeae* Mosauer, 1936	1	**GEN**	**VU**	–	**13**	**Pr**	**A**
*Lepidophyma micropholis* Walker, 1955	1	**SBT**	**VU**	?	**15**	**A**	**C/M**
*Lepidophyma occulor* Smith, 1942	1	**SBT**	**LC**	=	**14**	**Pr**	**A**
*Lepidophyma sylvaticum* Taylor, 1939	1	**SMO**	**LC**	–	**11**	**Pr**	**A**
**Xenosauridae (1 genus, 1 species)**							
*Xenosaurus newmanorum* Taylor, 1949	1	**SBT**	**EN**	–	**15**	**Pr**	**A**
**ORDER SQUAMATA**							
**SUBORDER SERPENTES**							
**Boidae (1 genus, 1 species)**							
*Boa imperator* Daudin, 1803	3	**SBT**	**NL**	?	**NE**	**NL**	**A**
**Colubridae (22 genera, 36 species)**							
*Arizona elegans* Kennicott, 1859	2	**CD**	**LC**	=	**5**	**NL**	**A**
*Coluber constrictor* Linnaeus, 1758	5	**SBT**	**LC**	=	**10**	**A**	**C/M**
*Conopsis lineata* (Kennicott, 1859)	1	**SMO**	**LC**	=	**13**	**NL**	**A**
*Conopsis nasus* Günther, 1858	1	**SMO**	**LC**	=	**11**	**NL**	**A**
*Drymarchon melanurus* (Duméril, Bibron, & Duméril, 1854)	4	**GEN**	**LC**	=	**6**	**NL**	**A**
*Drymobius chloroticus* (Cope, 1886)	3	**SBT**	**LC**	?	**8**	**NL**	**C/M**
*Drymobius margaritiferus* (Schlegel, 1837)	4	**GEN**	**NL**	?	**6**	**NL**	**A**
*Ficimia hardyi* Mendoza-Quijano & Smith, 1993	1	**CD**	**EN**	–	**13**	**NL**	**C/M**
*Ficimia olivacea* Gray, 1849	1	**SBT**	**NL**	?	**9**	**NL**	**C/M**
*Ficimia streckeri* Taylor, 1931	2	**GEN**	**LC**	=	**12**	**NL**	**C/M**
*Gyalopion canum* (Cope, 1861)	2	**CD**	**LC**	=	**9**	**NL**	**C/M**
*Lampropeltis annulata* Kennicott, 1861	2	**CD**	**NL**	?	**NE**	**NL**	**C/M**
*Lampropeltis mexicana* (Garman, 1884)	1	**CD**	**LC**	=	**15**	**A**	**A**
*Lampropeltis polyzona* Cope, 1860	1	**GEN**	**NL**	?	**11**	**NL**	**A**
*Lampropeltis splendida* (Baird & Girard, 1853)	2	**CD**	**NL**	?	**NE**	**NL**	**C/M**
*Leptophis mexicanus* Duméril & Bibron, 1854	3	**GEN**	**LC**	=	**6**	**A**	**A**
*Masticophis flagellum* (Shaw, 1802)	2	**CD**	**LC**	=	**8**	**A**	**A**
*Masticophis mentovarius* (Duméril, Bibron, & Duméril, 1854)	3	**GEN**	**LC**	?	**6**	**A**	**C/M**
*Masticophis schotti* Baird & Girard, 1853	2	**CD**	**LC**	=	**13**	**NL**	**A**
*Mastigodryas melanolomus* (Cope, 1868)	3	**SBT**	**LC**	=	**6**	**NL**	**C/M**
*Oxybelis aeneus* (Wagler, 1824)	4	**SBT**	**NL**	?	**5**	**NL**	**C/M**
*Pantherophis emoryi* (Baird & Girard, 1853)	2	**CD**	**LC**	=	**13**	**NL**	**A**
*Phrynonax poecilonotus* (Günther, 1858)	3	**SBT**	**NL**	?	**NE**	**NL**	**CM**
*Pituophis catenifer* Blainville, 1835	5	**GEN**	**LC**	=	**9**	**NL**	**A**
*Pituophis deppei* (Duméril, 1853)	1	**CD**	**LC**	=	**14**	**A**	**A**
*Pseudoelaphe flavirufa* (Cope, 1867)	3	**SBT**	**NL**	?	**10**	**NL**	**C/M**
*Rhinocheilus lecontei* Baird & Girard, 1853	2	**CD**	**LC**	=	**8**	**NL**	**A**
*Salvadora grahamiae* Baird & Girard, 1853	2	**CD**	**LC**	=	**10**	**NL**	**A**
*Senticolis triaspis* (Cope, 1866)	4	**GEN**	**LC**	=	**6**	**NL**	**A**
*Spilotes pullatus* (Linnaeus, 1758)	3	**SBT**	**NL**	?	**6**	**NL**	**A**
*Tantilla atriceps* (Günther, 1895)	2	**CD**	**LC**	=	**11**	**A**	**C/M**
*Tantilla bocourti* (Günther, 1895)	1	**CD**	**LC**	?	**9**	**NL**	**C/M**
*Tantilla rubra* Cope, 1876	3	**SBT**	**LC**	?	**5**	**Pr**	**C/M**
*Tantilla shawi* Taylor, 1949	1	**SBT**	**EN**	?	**15**	**Pr**	**C/M**
*Tantilla wilcoxi* Stejneger, 1902	2	**CD**	**LC**	=	**10**	**NL**	**C/M**
*Trimorphodon tau* Cope, 1870	1	**CD**	**LC**	?	**13**	**NL**	**C/M**
**Dipsadidae (14 genera, 22 species)**							
*Adelphicos quadrivirgatum* (Jan, 1862)	3	**SBT**	**LC**	?	**10**	**Pr**	**C/M**
*Amastridium sapperi* (Werner, 1903)		**SBT**	**LC**	=	**10**	**NL**	**Calzada-Arciniega (2014)**
*Chersodromus rubriventris* (Taylor, 1949)	1	**SBT**	**EN**	–	**14**	**Pr**	**C/M**
*Coniophanes fissidens* (Günther, 1858)	3	**SBT**	**NL**	?	**7**	**NL**	**C/M**
*Coniophanes imperialis* (Baird, 1859)	4	**SBT**	**LC**	=	**8**	**NL**	**C/M**
*Coniophanes piceivittis* Cope, 1869	3	**SBT**	**LC**	=	**7**	**NL**	**C/M**
*Diadophis punctatus* (Linnaeus, 1766)	5	**CD**	**LC**	=	**4**	**NL**	**A**
*Geophis latifrontalis* Garman, 1883	1	**CD**	**DD**	?	**14**	**Pr**	**C/M**
*Geophis mutitorques* (Cope, 1885)	1	**SBT**	**LC**	=	**13**	**Pr**	**C/M**
*Heterodon kennerlyi* Kennicott, 1860	2	**CD**	**NL**	?	**11**	**NL**	**C/M**
*Hypsiglena jani* (Dugès, 1865)	2	**CD**	**NL**	?	**6**	**NL**	**A**
*Hypsiglena tanzeri* Dixon & Lieb, 1972	1	**CD**	**DD**	?	**15**	**NL**	**C/M**
*Imantodes cenchoa* (Linnaeus, 1758)	3	**SBT**	**NL**	?	**6**	**Pr**	**A**
*Leptodeira maculata* (Linnaeus, 1758)	1	**GEN**	**LC**	=	**7**	**Pr**	**A**
*Leptodeira septentrionalis* (Kennicott, 1859)	3	**GEN**	**NL**	?	**8**	**NL**	**C/M**
*Ninia diademata* Baird & Girard, 1853	3	**SBT**	**LC**	=	**9**	**NL**	**A**
*Pliocercus elapoides* Cope, 1860	3	**SBT**	**LC**	=	**10**	**NL**	**A**
*Rhadinaea decorata* (Günther, 1858)	3	**SBT**	**NL**	?	**9**	**NL**	**A**
*Rhadinaea gaigeae* Bailey, 1937	1	**GEN**	**DD**	?	**12**	**NL**	**A**
*Rhadinaea marcellae* Taylor, 1949	1	**SBT**	**EN**	–	**12**	**Pr**	**C/M**
*Tropidodipsas fasciata* Günther, 1858	3	**SBT**	**NL**	?	**13**	**NL**	**C/M**
*Tropidodipsas sartorii* Cope, 1863	3	**GEN**	**LC**	=	**9**	**Pr**	**A**
**Elapidae (1 genus, 1 species)**							
*Micrurus tener* Baird & Girard, 1853	2	**GEN**	**LC**	=	**11**	**NL**	**A**
**Leptotyphlopidae (1 genus, 3 species)**							
*Rena dulcis* Baird & Girard, 1853	2	**GEN**	**LC**	?	**13**	**NL**	**C/M**
*Rena myopica* (Garman, 1884)	1	**GEN**	**LC**	=	**13**	**NL**	**C/M**
*Rena segrega* (Klauber, 1939)	2	**CD**	**NL**	?	**NE**	**NL**	**C/M**
**Natricidae (3 genera, 12 species)**							
*Nerodia rhombifer* (Hallowell, 1852)	2	**GEN**	**LC**	=	**10**	**NL**	**C/M**
*Storeria dekayi* (Holbrook, 1939)	5	**SMO**	**LC**	=	**7**	**NL**	**A**
*Storeria hidalgoensis* Taylor, 1942	1	**SMO**	**VU**	–	**13**	**NL**	**A**
*Storeria storerioides* (Cope, 1866)	1	**SMO**	**LC**	=	**11**	**NL**	**A**
*Thamnophis cyrtopsis* (Kennicott, 1860)	4	**GEN**	**LC**	=	**7**	**A**	**A**
*Thamnophis eques* (Reuss, 1834)	2	**GEN**	**LC**	=	**8**	**A**	**A**
*Thamnophis marcianus* (Baird & Girard, 1853)	4	**GEN**	**LC**	?	**10**	**A**	**A**
*Thamnophis melanogaster* (Wiegmann, 1830)	1	**CD**	**EN**	–	**15**	**A**	**A**
*Thamnophis proximus* (Say, 1823)	4	**SBT**	**LC**	=	**7**	**A**	**C/M**
*Thamnophis scalaris* Cope, 1861	1	**SMO**	**LC**	=	**14**	**A**	**C/M**
*Thamnophis scaliger* (Jan, 1863)	1	**SMO**	**VU**	–	**15**	**A**	**C/M**
*Thamnophis sumichrasti* (Cope, 1866)	1	**SMO**	**LC**	?	**15**	**A**	**A**
**Viperidae (4 genera, 10 species)**							
*Agkistrodon taylori* Burger & Robertson, 1951	1	**SMO**	**LC**	?	**17**	**A**	**Lemos-Espinal and Dixon (2013)**
*Atropoides nummifer* (Rüppell, 1845)	1	**SBT**	**LC**	=	**13**	**A**	**Lemos-Espinal and Dixon (2013)**
*Bothrops asper* (Garman, 1883)	3	**SBT**	**NL**	?	**12**	**NL**	**Lemos-Espinal and Dixon (2013)**
*Crotalus aquilus* Klauber, 1952	1	**GEN**	**LC**	–	**16**	**Pr**	**A**
*Crotalus atrox* Baird & Girard, 1853	2	**CD**	**LC**	=	**9**	**Pr**	**A**
*Crotalus lepidus* (Kennicott, 1861)	2	**CD**	**LC**	=	**12**	**Pr**	**A**
*Crotalus molossus* Baird & Girard, 1853	2	**CD**	**LC**	=	**8**	**Pr**	**A**
*Crotalus pricei* Van Denburgh, 1895	2	**SMO**	**LC**	=	**14**	**Pr**	**Lemos-Espinal and Dixon (2013)**
*Crotalus scutulatus* (Kennicott, 1861)	2	**CD**	**LC**	=	**11**	**Pr**	**A**
*Crotalus totonacus* Gloyd & Kauffeld, 1940	1	**SBT**	**NL**	?	**17**	**NL**	**A**

**Table 2.** List of amphibian and reptile species that potentially occur in San Luis Potosí.

CLASS AMPHIBIA	
**ORDER CAUDATA**	
**Plethodontidae**	
*Aquiloeurycea cephalica* (Cope, 1869)	Likely to occur in south-southeastern SLP (credible but not documented or published records exist)
*Bolitoglossa rufescens* (Cope, 1869)	Reported by Taylor (1949) without museum record
**ORDER ANURA**	
**Bufonidae**	
*Incilius marmoreus* (Wiegmann, 1833)	Likely to occur in southeastern SLP
*Incilius valliceps* (Wiegmann, 1833)	Likely to occur in southeastern SLP
**Craugastoridae**	
*Craugastor rhodopis* (Cope, 1867)	Reported by Taylor (1949) without museum record
**Eleutherodactylidae**	
*Eleutherodactylus dennisi* (Lynch, 1970)	Likely to occur in southeastern SLP
*Eleutherodactylus nitidus* (Peters, 1870)	Likely to occur in southeastern SLP
**Hylidae**	
*Sarcohyla bistincta* (Cope, 1877)	Likely to occur in southeastern SLP
**Ranidae**	
*Rana spectabilis* Hillis & Frost, 1985	Likely to occur in southeastern SLP
**CLASS REPTILIA**	
**ORDER TESTUDINES**	
**Testudinidae**	
*Gopherus berlandieri* (Agassiz, 1857)	Reported by Taylor (1949) without museum record
**ORDER SQUAMATA**	
**SUBORDER LACERTILIA**	
**Anguidae**	
*Gerrhonotus farri* Bryson & Graham, 2010	Likely to occur in central-eastern SLP
**Phrynosomatidae**	
*Sceloporus aeneus* Wiegmann, 1828	Likely to occur in southeastern SLP
**Scincidae**	
*Scincella gemmingeri* (Cope, 1864)	Likely to occur in southeastern SLP
**Teiidae**	
*Aspidoscelis neomexicanus* (Lowe & Zweifel, 1952)	Reported by Taylor (1949) without museum record
**ORDER SQUAMATA**	
**SUBORDER SERPENTES**	
**Colubridae**	
*Lampropeltis ruthveni* Blanchard, 1920	Likely to occur in southern SLP
**Dipsadidae**	
*Rhadinaea montana* Smith, 1944	Likely to occur in central-eastern SLP
**Leptotyphlopidae**	
*Epictia goudotti* (Duméril & Bibron, 1844)	Likely to occur in southeastern SLP

### Habitat types

When considering all the species of amphibians and reptiles in San Luis Potosí, the number of species in the Chihuahuan Desert, the subtropics of the Sierra Madre Oriental, and generalist habitat types are about equal with 30% of the species occurring in each of these habitat types (Table 3) The temperate forests of the Sierra Madre Oriental has much fewer species (Table 3); however, this overall pattern is primarily a function of the distribution of reptile species, since all reptile groups tend to follow this pattern, with the number of reptile species found only in the temperate forests of the Sierra Madre Oriental being particularly low compared to the other habitat types (Table 3). For amphibians, the pattern is more complicated. Anurans have a higher number of species using the subtropics of the Sierra Madre Oriental and are generalists more than either the Chihuahuan Desert and the temperate forests of the Sierra Madre Oriental (Table 3). On the other hand, 50% of salamander species are found in the temperate forests of the Sierra Madre Oriental (Table 3). These patterns of distribution for amphibians likely parallel their need for moist habitats.

At the family level, some families appear to be primarily associated with specific habitat types whereas others are found across habitat types. Bufonidae, Phrynosomatidae, and Teiidae are primarily associated with the Chihuahuan Desert; Plethodontidae is primarily found in the Sierra Madre Oriental; Hylidae is primarily found in the subtropics of the Sierra Madre Oriental; Colubridae and Dipsadidae are often found in the Chihuahuan Desert and the subtropics of the Sierra Madre Oriental, but few of their species are found in the Sierra Madre Oriental; and Viperidae are found in all three habitat types.

**Table 3.** Summary of the number of native species (% in parentheses) in different taxonomic groups found in different habitat types in San Luis Potosí, Mexico (see Table 2 for abbreviations).

Taxa	CD	SMO	SBT	GEN
Amphibia	7 (17.5)	7 (17.5)	13 (32.5)	13 (32.5)
Caudata	1 (16.7)	3 (50)	2 (33.3)	0 (0)
Anura	6 (17.6)	4 (11.8)	11 (32.4)	13 (32.5)
Reptilia	48 (34.8)	12 (8.7)	37 (26.8)	41 (29.7)
Crocodylia	0 (0)	0 (0)	1 (100)	0 (0)
Testudines	0 (0)	0 (0)	3 (42.8)	4 (57.1)
Squamata	48 (36.9)	12 (9.2)	33 (25.4)	37 (28.5)
Lacertilia	16 (35.6)	2 (4.4)	10 (22.2)	17 (37.8)
Serpentes	32 (37.6)	10 (11.8)	23 (27.0)	20 (23.5)
Total	55 (30.9)	19 (10.7)	50 (28.1)	54 (30.3)

### Comparisons with neighboring states

**Figure 5.**
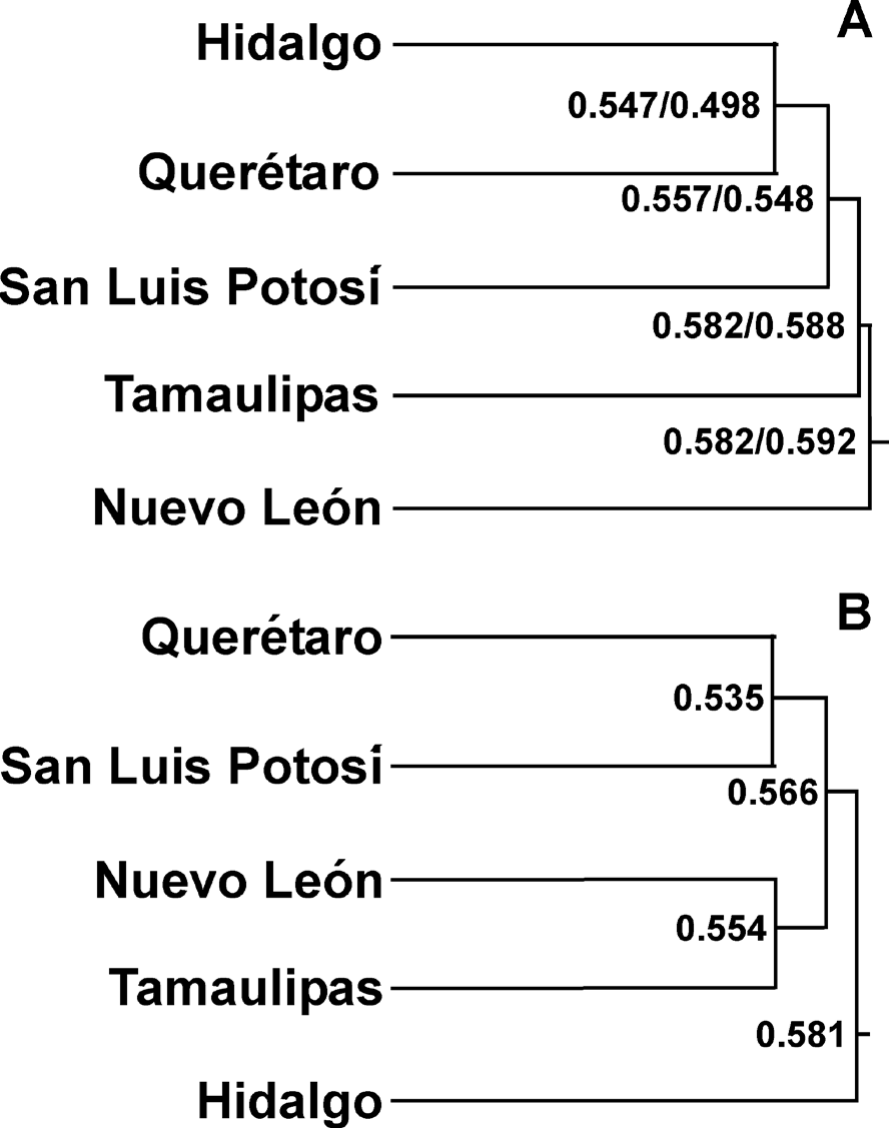
Results of cluster analysis of the herpetofaunas of San Luis Potosí and its neighboring states (Hidalgo, Nuevo León, Querétaro, and Tamaulipas). The distances provided are Euclidean distances for **A** the entire herpetofauna and reptiles only, respectively and **B** amphibians only.

Overall, San Luis Potosí shares the most species with Hidalgo and Tamaulipas, and shares the least number of species with Nuevo León (Table 4). It is particularly interesting to note that for most taxa Hidalgo shares the highest proportion of species with San Luis Potosí whereas Tamaulipas shares the highest proportion of lizard species by a large margin and Nuevo León shares a very high proportion (≈ 90%) of phrynosomatid species (Table 4). It is likely that this reflects the more arid nature of Tamaulipas and Nuevo León (i.e., Chihuahuan Desert), compared to the more mountainous Querétaro and Hidalgo. Thus, the numbers and types of shared species among San Luis Potosí and its neighboring states reflects the pattern of habitat and vegetation types found in each neighboring state (see also Smith and Lemos-Espinal 2015, Lemos-Espinal and Smith 2016, Lemos-Espinal et al. 2017). However, the results of the cluster analysis are somewhat different. The cluster analysis found that San Luis Potosí is clustered with the pair of Hidalgo and Querétaro for all species together and reptiles (Figure 5A). In contrast, for amphibians San Luis Potosí clusters with Querétaro, and this pair clusters with the pair of Nuevo León and Hidalgo (Figure 5B). Thus, it appears that amphibians and reptiles show different affinities among these states, again perhaps reflecting the available habitats or environments in each state. It therefore appears that in addition to state-specific conservation and management plans, more integrated habitat specific conservation plans that allow inter-state efforts would be the best approach to preserve the herpetofauna of San Luis Potosí and its neighboring states. In addition, the results of the cluster analysis suggest that amphibians and reptiles will each require different inter-state collaborations (i.e., the states involved in such collaborations might differ between amphibians and reptiles based on the different patterns of clustering between these taxa).

**Table 4.** Summary of the numbers of species shared between San Luis Potosí and neighboring Mexican states (not including introduced species). The percent of species from San Luis Potosí shared by a neighboring state are given in parentheses. – indicates either San Luis Potosí or the neighboring state has no species in the taxonomic group, thus no value for shared species is provided.

Taxa	San Luis Potosí	Hidalgo	Querétaro	Nuevo León	Tamaulipas
**Class Amphibia**	40	35 (87.5)	26 (65)	17 (42.5)	29 (72.5)
**Order Caudata**	6	5 (83.3)	4 (66.7)	0 (0)	4 (66.7)
Ambystomatidae	1	1 (100)	1 (100)	0 (0)	–
Plethodontidae	4	3 (75)	3 (75)	0 (0)	3 (75)
Salamandridae	1	1 (100)	–	–	1 (100)
**Order Anura**	34	30 (88.2)	22 (64.7)	17 (52)	25 (73.5)
Bufonidae	6	4 (67)	4 (67)	5 (83.3)	5 (83.3)
Craugastoridae	3	3 (100)	2 (67)	1 (33)	2 (67)
Eleutherodactylidae	5	3 (60)	3 (60)	3 (60)	4 (80)
Hylidae	9	9 (100)	6 (67)	2 (22.2)	5 (55.6)
Leptodactylidae	2	2 (100)	–	1 (50)	2 (100)
Microhylidae	2	1 (50)	1 (50)	2 (100)	2 (100)
Ranidae	4	4 (100)	3 (75)	1 (25)	2 (50)
Rhinophrynidae	1	1 (100)	1 (100)	–	1 (100)
Scaphiopodidae	2	2 (100)	2 (100)	2 (100)	2 (100)
**Class Reptilia**	138	98 (71.0)	92 (66.7)	75 (54.3)	100 (72.5)
**Order Crocodylia**	1	1 (100)	0 (0)	0 (0)	1 (100)
Crocodylidae	1	1 (100)	–	–	1 (100)
**Order Testudines**	7	5 (71.4)	3 (42.8)	2 (28.6)	5 (71.4)
Emydidae	2	1 (50)	–	0 (0)	1 (50)
Kinosternidae	4	4 (100)	3 (75)	1 (25)	3 (75)
Trionychidae	1	–	–	1 (100)	1 (100)
**Order Squamata**	130	92 (70.8)	73 (56.2)	73 (56.2)	94 (72.3)
**Suborder Lacertilia**	45	25 (55.6)	22 (48.9)	27 (60.0)	36 (80.0)
Anguidae	5	3 (60)	1 (20)	2 (40)	5 (100)
Corytophanidae	2	1 (50)	1 (50)	–	1 (50)
Crotaphytidae	1	–	–	1 (100)	1 (100)
Dactyloidae	2	2 (100)	1 (50)	–	1 (50)
Dibamidae	1	1 (100)	1 (100)	–	1 (100)
Eublepharidae	1	–	–	–	–
Iguanidae	1	1 (100)	–	–	1 (100)
Phrynosomatidae	19	9 (47.4)	10 (52.6)	17 (89.5)	15 (78.9)
Scincidae	5	3 (60)	3 (60)	4 (80)	5 (100)
Teiidae	3	2 (66.7)	2 (66.7)	2 (66.7)	3 (100)
Xantusiidae	4	2 (50)	3 (75)	1 (25)	2 (50)
Xenosauridae	1	1 (100)	0 (0)	–	1 (100)
**Suborder Serpentes**	85	67 (78.8)	51 (60.0)	46 (54.1)	58 (68.2)
Boidae	1	1 (100)	1 (100)	–	1 (100)
Colubridae	36	24 (66.7)	22 (61.1)	23 (63.9)	27 (75)
Dipsadidae	22	19 (86.4)	13 (59.1)	6 (27.3)	14 (63.6)
Elapidae	1	1 (100)	1 (100)	1 (100)	1 (100)
Leptotyphlopidae	3	2 (66.7)	1 (33.3)	2 (66.7)	2 (66.7)
Natricidae	12	12 (100)	7 (58.3)	7 (58.3)	6 (50)
Viperidae	10	8 (80)	6 (60)	7 (70)	7 (70)
**TOTAL**	**178**	**133 (74.8)**	**118 (66.3)**	**92 (51.7)**	**129 (72.5)**

### Conservation status

**Figure 6.**
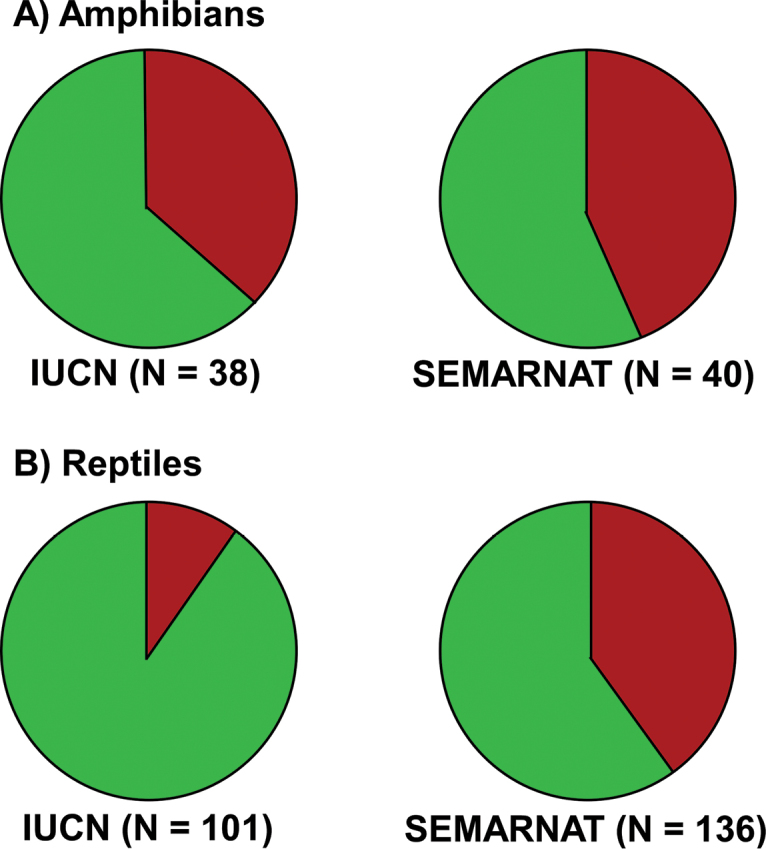
Percent of **A** amphibians and **B** reptiles listed in protected categories on the IUCN Red List and SEMARNAT. Green is percent in Least Concern (IUCN) or Not Listed (SEMARNAT), Red is percent in protected categories. N is the total number of species assessed by each agency.

Nearly 82% of the amphibians and reptile species that have been evaluated by the IUCN falls in the Least Concern category (does not include DD species; Table 5, Figure 6). However, only 60% are not listed by SEMARNAT (Table 5, Figure 6). The discrepancy between the IUCN and SEMARNAT listings are greater for reptiles than amphibians (Figure 6). The average EVS for all herpetofaunal species in San Luis Potosí that have been evaluated is 10.67. These overall numbers tend to obscure the presence of particular groups, or even individual species, that occur in San Luis Potosí that are at potential risk and that may warrant special attention. Some taxa of particular concern, based on their IUCN listing, SEMARNAT category, or their EVS include the salamanders in general, and Plethodontidae and Salamandridae in particular; the emydid and trionychid turtles, anguid and xenosaurid lizards, and natricid and colubrid snakes. These taxa reflect assessments at the global or country-level scale. It may be, and indeed it is likely, that there are multiple species of amphibians and reptiles that are more or less threatened at the state level than these larger scale assessments suggest. However, given the relative paucity of population-level studies and assessments on the herpetofauna of San Luis Potosí, such conservation or management needs are unknown.

**Table 5.** Summary of native species present in San Luis Potosí by family, order or suborder, and class. Status summary indicates the number of species found in each IUCN conservation status in the order DD, LC, V, NT, E, CE (see Table 1 for abbreviations; in some cases species have not been assigned a status by the IUCN and therefore these may not add up to the total number of species in a taxon). Mean EVS is the mean Environmental Vulnerability Score, scores > 14 are considered high vulnerability (Wilson et al., 2013a,b) and conservation status in Mexico according to SEMARNAT (2010) in the order NL, Pr, A, P (see Table 1 for abbreviations).

Class	Order/ Suborder	Family	Status Summary	Mean EVS	SEMARNAT
Amphibia	Caudata		0,1,1,1,1,1	13.33	0,4,1,1
		Ambystomatidae	0,1,0,0,0,0	10	0,1,0,0
		Plethodontidae	0,0,1,1,1,1	14.5	0,3,1,0
		Salamandridae	0,0,0,0,1,0	12	0,0,0,1
	Anura		0,24,4,3,2,0	8.93	22,9,2,1
		Bufonidae	0,5,0,0,0,0	7.6	5,1,0,0
		Craugastoridae	0,1,1,1,0,0	12.33	1,2,0,0
		Eleutherodactylidae	0,2,3,0,0,0	13.2	4,1,0,0
		Hylidae	0,7,0,1,1,0	7.44	7,1,1,0
		Leptodactylidae	0,2,0,0,0,0	5.5	2,0,0,0
		Microhylidae	0,2,0,0,0,0	6.5	1,1,0,0
		Ranidae	0,2,0,1,1,0	11.75	0,2,1,1
		Rhynophrynidae	0,1,0,0,0,0	8	0,1,0,0
		Scaphiopodidae	0,2,0,0,0,0	4.5	2,0,0,0
	**Subtotal**		0,25,5,4,3,1	9.59	22,13,3,2
Reptilia	Crocodylia		0,1,0,0,0,0	13	0,1,0,0
		Crocodylidae	0,1,0,0,0,0	13	0,1,0,0
	Testudines		0,3,0,1,0,0	13.14	2,5,0,0
		Emydidae	0,0,0,0,0,0	16	2,0,0,0
		Kinosternidae	0,2,0,1,0,0	11.25	0,4,0,0
		Trionychidae	0,1,0,0,0,0	15	0,1,0,0
	Squamata		4,84,5,0,7,0	10.82	80,26,22,1
	Lacertilia		1,29,3,0,2,0	11.84	28,10,6,1
		Anguidae	1,2,1,0,0,0	14	3,1,0,1
		Corytophanidae	0,1,0,0,0,0	10.5	0,2,0,0
		Crotaphytidae	0,1,0,0,0,0	13	0,0,1,0
		Dactyloidae	0,0,0,0,0,0	8.5	2,0,0,0
		Dibamidae	0,1,0,0,0,0	10	0,0,1,0
		Eublepharidae	0,1,0,0,0,0	9	0,0,1,0
		Iguanidae	0,0,0,0,0,0	12	0,1,0,0
		Phrynosomatidae	0,16,0,0,1,0	12	16,1,2,0
		Scincidae	0,3,0,0,0,0	10.0	4,1,0,0
		Teiidae	0,2,0,0,0,0	11.7	3,0,0,0
		Xantusiidae	0,2,2,0,0,0	13.25	0,3,1,0
		Xenosauridae	0,0,0,0,1,0	15	0,1,0,0
	Serpentes		3,55,2,0,5,0	10.29	52,16,16,0
		Boidae	0,0,0,0,0,0		1,0,0,0
		Colubridae	0,25,0,0,2,0	9.42	27,2,6,0
		Dipsadidae	3,10,0,0,2,0	9.73	14,8,0,0
		Elapidae	0,1,0,0,0,0	11	1,0,0,0
		Leptotyphlopidae	0,2,0,0,0,0	13	3,0,0,0
		Natricidae	0,9,2,0,1,0	11	4,0,8,0
		Viperidae	0,8,0,0,0,0	12,9	2,6,2,0
	**Subtotal**		4,88,5,1,7,0	10.95	82,32,22,1
TOTAL			4,109,10,4,10,1	10.64	105,45,25,3

The conservation status of the reptiles and amphibians in each habitat type was examined. For amphibians, the percentage of species in protected IUCN categories (VU, NT, EN, CE) varied among the habitat types. Twenty-nine percent of amphibians in the Chihuahuan Desert were listed in IUCN categories, 72% in the Sierra Madre Oriental, 46% in the subtropics of the Sierra Madre Oriental, and 8% of the generalists. For SEMARNAT categories, 57% of amphibians in the Chihuahuan Desert, 72% in the Sierra Madre Oriental, 46% of the subtropics of the Sierra Madre Oriental, and 23% of the generalists were listed. Thus, for amphibians, species found in the Sierra Madre Oriental are the most threatened whereas the generalists were least threatened. Reptiles showed a slightly different pattern. For the IUCN listings, all habitat types had relatively few species in the protected categories (Chihuahuan Desert, 8%; Sierra Madre Oriental, 18%; subtropics of the Sierra Madre Oriental, 10%; and generalists, 9%). However, for SEMARNAT, 28% of reptiles in the Chihuahuan Desert, 50% from the Sierra Madre Oriental, 50% from the subtropics of the Sierra Madre Oriental, and 42% of the generalist species were in the protected categories. For reptiles, the conservation status of the species in each habitat type is more evenly distributed across the habitat types than in amphibians.

Hopefully, by establishing this list of herpetofaunal species with their global and country-level conservation statuses will prompt further investigations into the amphibians and reptiles of this state, which could provide the needed information to allow for state specific, or even habitat type, conservation measures to be undertaken. Specific threats known to be present in San Luis Potosí are deforestation and habitat loss (Miranda-Aragón et al. 2012, Reyes Hernández et al. 2013, Ramos-Lara and Koprowski 2014), industrial pollutants and heavy metals (Alcalá-Jáuregui et al. 2014, Pérez-Vázquez et al. 2016a, b), mining (Razo et al. 2004, Chapa-Vargas et al. 2010, Espinosa-Reyes et al. 2014), and overexploitation of water resources (Esteller et al. 2012).
